# Association Between Inflammatory Biomarkers and Mental Health Symptoms in Middle Eastern Refugees in the US

**DOI:** 10.7759/cureus.28246

**Published:** 2022-08-21

**Authors:** Malek Ghandour, Jolin B Yamin, Judith E Arnetz, Mark A Lumley, Paul M Stemmer, Paul Burghardt, Hikmet Jamil, Bengt B Arnetz

**Affiliations:** 1 Department of Obstetrics and Gynecology, Wayne State University, Detroit, USA; 2 Department of Psychology, Wayne State University, Detroit, USA; 3 Department of Family Medicine, Michigan State University, East Lansing, USA; 4 Department of Pharmaceutical Sciences, Wayne State University, Detroit, USA; 5 Department of Nutrition & Food Science, Wayne State University, Detroit, USA; 6 Department of Family Medicine, Wayne State University, Detroit, USA

**Keywords:** refugees, biomarkers, war, depression, anxiety

## Abstract

Introduction: Refugees are at increased risk for trauma-related mental health disorders, including anxiety, depression, and post-traumatic stress disorder (PTSD). The underlying biological mechanisms linking trauma to mental disorders need additional study, and the possible pathophysiological role of the immune system is attracting increasing interest. In this study, we investigated whether two well-known pro-inflammatory cytokines (interleukin (IL-8) and IL-6) are associated with mental health symptoms in Middle Eastern refugees displaced to the United States.

Methods: Refugees (n=64, mean age=37.6 years) ages ranged from 21 to 74 years (mean=37.62, SD=11.84) were interviewed one month after arrival in Michigan, United States, using a validated survey in Arabic. Questions covered pre-displacement trauma, current anxiety, depression, and PTSD symptoms. Blood, collected immediately following the interview, was analyzed for the levels of interleukins. Multivariate linear regression was used to determine the association between mental health symptoms and IL-6 and IL-8.

Results: In multivariate modeling, older age (β=0.37; p<0.01) and anxiety (β=0.31; p<0.05) were positively associated with IL-8. Age (β=0.28; p<0.05) and pre-displacement trauma (β=0.40; p<0.05) were positively associated with IL-6. Depression (β=-0.38) was negatively associated with IL-6.

Conclusion/relevance: This study of inflammatory biomarkers suggests the possibility of differential associations between mental health symptoms (anxiety and depression) and pro-inflammatory markers (IL-6 and IL-8). To enhance our ability to prevent and more effectively treat trauma-exposed refugees, we need to better understand the neuroinflammatory mechanisms contributing to mental disorders.

## Introduction

The relationship between war- or trauma-associated exposures and adverse mental health is well established [1-5]. Although exposure to trauma is necessary to develop trauma-associated mental health disorders, exposure alone is not sufficient [3]. For example, with respect to developing post-traumatic stress disorder (PTSD), both the intensity of trauma exposure and the type of exposure are important [6]. There is, therefore, an increasing interest in risk and protective factors that might moderate the direct impact of stress and war-related trauma exposures on mental health. Research suggests that genetics, gender, adverse childhood experiences (ACEs), and lower intellectual processing capacity are moderators [7-11]. Moreover, Arnetz et al. found that only two of the five subscales of the commonly used war-related trauma exposure assessment instrument, the Harvard Trauma Questionnaire (HTQ), “physical trauma to self” and “lack of necessities”, predicted PTSD in a large, prospective study of Middle Eastern refugees displaced to the United States [6].

Alterations in inflammatory systems are common in several trauma/stress-associated mental health symptoms and disorders, including PTSD, depression, and anxiety [12-17]. Inflammatory cytokines, such as interleukin-6 (IL-6) and interleukin 8 (IL-8), have demonstrated various associations with mental health and neuropsychiatric symptoms [12-16]. IL-6 is an inflammatory biomarker that causes oxidative stress in the brain [13]. Recent publications, including meta-analyses, report that IL-6 is elevated in people with PTSD compared to healthy controls [15,18]. Another study found a positive association between IL-6 and depressive symptoms [19]. In a prospective study, mentally healthy nine-year-olds with elevated IL-6 were more likely to be diagnosed with depression at the age of 18 years, compared to children without elevated IL-6 [13].

IL-8 is a pro-inflammatory cytokine produced by many cell types including macrophages and microglia. In the brain, IL-8 is released from microglia in response to pro-inflammatory stimuli [20]. One study reported higher levels of IL-8 in the cerebrospinal fluid (CSF) of patients diagnosed with unipolar depression as compared to healthy controls [21]. In another study, there was a significant inverse association between IL-8 levels and anxiety in suicide attempters [14]. In contrast, there was no difference in IL-8 between healthy controls and people with major depressive disorder (MDD) [14]. Another study reported that a diagnosis of PTSD was associated with lower levels of IL-8; however, this study observed a positive association between anxiety and depression symptom severity and IL-6 levels in earthquake survivors [15]. Thus, it is possible that both IL-6 and IL-8, although pro-inflammatory, play different, possibly opposite roles in certain trauma-induced mental health disorders.

There are several possible reasons for these inconsistencies across studies and trauma-associated symptoms, including variation in participants' age, gender, pre-morbid vulnerability, genetic and epigenetic characteristics, geography, and environmental conditions [4-5,10-11,13,15,18,22,23]. The type and intensity of trauma or triggers also vary among studies as does the use of different diagnostic evaluation tools and criteria for psychiatric diagnoses [6,14,15,21]. In summary, to further delineate the relationship among trauma, mental health, and inflammatory markers, there is a need for more comprehensive studies where validated instruments and methods are used to characterize participants’ mental health, prior trauma exposures, and the sensitivity and accuracy of biomarker assessment methods.

Focusing on the role of specific inflammatory biomarkers might enhance our understanding of risk for mental health problems, resulting in an increased capability to identify risk groups and fine-tune primary prevention, treatment, and relapse prevention strategies. In the current cross-sectional study, we studied the association between war-related trauma exposure, specific inflammatory markers, and symptoms of depression, anxiety, and PTSD.

The two specific aims were as follows: Aim 1: To determine the association between war-related trauma exposure and the pro-inflammatory cytokines IL-6 and IL-8, we hypothesized that higher war-related trauma exposures would be associated with higher serum levels of both IL-6 and IL-8. Aim 2: To determine the association between symptoms of anxiety, depression, and PTSD symptom scores and the two inflammatory biomarkers, we hypothesized that depression, anxiety, and PTSD would be positively associated with IL-6 and IL-8. This article was previously presented as a poster at the American College of Physicians (ACP) Michigan Chapter Annual Fall Scientific Meeting on October 12, 2019, and the Virtual Michigan State University/Flint Area Medical Education (MSU-FAME) Research Forum on May 2020.

## Materials and methods

Recruitment and consenting

The study group consisted of 64 Iraqi and Syrian refugees, who had arrived in Detroit, Michigan, within the prior month. Information about the research study was provided during refugee orientation sessions offered by the Samaritas Social Service Organization (SSSO), a refugee resettlement agency. These sessions were part of the basic resettlement information process. At these sessions, the intention was not to recruit study participants. Rather, the newly arrived refugees were informed by the SSSO staff that the study team was interested to have potential study participants contact them to receive more information. To respect refugees’ vulnerable situations and avoid any actual or perceived pressure to agree to participate, the research team did not directly provide study information. Rather, the recruitment process required that potential participants reported an interest to SSSO for further information about the study directly from the research team. The SSSO then informed interested refugees to contact the research team directly to arrange a time for a meeting to receive additional information from a member of the research team. At that meeting, potential study participants received detailed oral and written information about the study, in Arabic, and were provided an opportunity to ask questions and have them answered. Those interested in enrolling in the study signed a consent form written in Arabic.

Data collection

Refugees who consented to participate were scheduled for an individual meeting with a research team member approximately one month after their arrival in the United States. The meeting took place at the Arab Community Center for Economic and Social Services (ACCESS), which is a non-profit organization designed to offer social, economic, health, and educational services for the Arab Community and beyond. An Arabic-speaking research assistant used a previously validated, structured survey in Arabic when interviewing participants [6]. All interviews were done in a private room at the ACCESS facility, and participants were provided transportation free of charge to get there if necessary. The survey contained questions about socioeconomic status (SES) and demographics. Depressive symptom scores were assessed using the Hospital Anxiety and Depression Scale (HADS) and trauma symptoms using the PTSD Checklist-C (PLC-C) [6,24]. The Harvard Trauma Questionnaire (HTQ) was used to quantify pre-displacement trauma exposure to 39 potentially traumatic events specific to refugee groups [6]. Anxiety symptoms were assessed using the validated Beck Anxiety Inventory (BAI) [25].

Immediately following the interview, blood samples were collected by a phlebotomist. Specimens were collected between 12:00 PM and 3:30 PM to reduce the influence of circadian rhythms. Serum was prepared and frozen for later analysis to determine the concentration of IL-6 and IL-8 using multiplex ELISA (Mesoscale Discovery Platform). Although the study had 64 consented participants, complete data were available for only 60 participants. The Wayne State University (WSU) institutional review board (IRB) and the Michigan State University (MSU) IRB approved this study (IRB numbers: 1506014063 and #15-951, respectively).

Statistical analyses

Data were analyzed using IBM SPSS statistics, V.28, 2022 (IBM Corp, Armonk, NY). Serum levels of both IL-6 and IL-8 were skewed, so they were log-transformed. Bivariate analysis was used to determine correlations among the two inflammatory markers, trauma exposure, and three symptom measures. Linear regression was used to investigate the association between each of the mental health symptoms (anxiety, depression, and PTSD scores) and IL-6 and IL-8, respectively. In the linear regression modeling, we controlled for age and gender given that both variables, but especially age, are related to systemic inflammation [22,23]. A two-sided p-value of <0.05 represents statistical significance.

## Results

Table 1 depicts the characteristics of the study participants. The age ranged from 21 to 74 years. About half of the participants self-identified as females and the rest as males. A larger proportion of the participants were from Syria (62.5%) than from Iraq.

**Table 1:** Characteristics of questionnaire respondents (n=64)

Age, years, mean (SD)	37.6 (11.78)
Gender, n (%)	
Male	33 (51.6)
Female	31 (48.4)
Country of birth, n (%)	
Syria	40 (62.5)
Iraq	24 (37.5)
Tobacco consumption in the last 30 days, n (%)	
Current smoker	25 (39.1)
Former smoker	3 (4.7)
Never smoked	36 (56.3)

Table 2 reports bivariate correlations among pre-displacement trauma scores, mental health symptoms, and inflammatory biomarkers. Depression, anxiety, and PTSD scores were all positively correlated with each other, whereas the two biomarkers had a positive but non-significant relationship with each other. Mental health symptoms were positively correlated with pre-displacement trauma. Most of the correlations of mental health symptoms with IL-6 and IL-8 were small in magnitude but did not reach statistical significance.

**Table 2:** Bivariate correlation between trauma, mental health disorders, and inflammatory biomarkers (n=64) ***p<0.001. pg/ml, picograms per milliliter; α, Cronbach’s alpha.

Bivariate correlation of variables 1-6 with corresponding labels							
	Mean (SD)	1	2	3	4	5	6
1. War-related trauma exposure (α=0.87)	12.09 (6.02)	-					
2. Depression (α=0.86)	6.31 (4.35)	0.50***	-				
3. Anxiety; 0-63 (α=0.92)	13.02 (11.30)	0.48***	0.63***	-			
4. Post-traumatic stress disorder (α=0.82)	33.95 (12.41)	0.48***	0.64***	0.67***	-		
5. Interleukin-6 (pg/ml)	1.24 (2.98)	0.15	-0.18	0.2	0.02	-	
6. Interleukin-8 (pg/ml)	3.92 (2.46)	-0.02	0.14	0.25	0.16	0.19	-

Table 3 depicts results for the linear regression model predicting IL-6 levels. In the full model, age (β=0.28, p<0.05) and war-related trauma exposure (β=0.40, p<0.05) were significantly positively associated with IL-6 levels. Depression was negatively associated with IL-6 levels (β=-0.38, p<0.05). The model explained 19% of the overall variance in IL-6.

**Table 3:** Predicting interleukin-6 in refugees using linear regression (n=64) *p<0.05. β, standardized beta.

	Step 1	Step 2	Step 3
	β	β	β
Age	0.24	0.24	0.28*
Gender	0.05	0.11	-0.1
War-related trauma exposure		0.17	0.40*
Depression			-0.38*
R^2^	0.06	0.09	0.19

Table 4 depicts predictors of IL-8 using linear regression modeling. Age (standardized β=0.37; p<0.01) was positively associated with IL-8 levels. After controlling for age, gender, and war-related trauma exposure, anxiety (β=0.31; p<0.05) was positively associated with IL-8. War-related trauma exposure and gender were not statistically significantly associated with IL-8 levels. The model explained 24% of the overall variance.

**Table 4:** Predicting interleukin-8 in refugees using linear regression (n=64) *p<0.05, **p<0.01. β, standardized beta.

	Step 1	Step 2	Step 3
	β	β	β
Age	0.41**	0.41**	0.37**
Gender	-0.01	-0.02	-0.1
War-related trauma exposure		-0.04	-0.21
Anxiety			0.31*
R^2^	0.17	0.17	0.24

Other pertinent data regarding PTSD were noted. After controlling for age, gender, and war-related trauma exposure, PTSD was not associated with either IL-6 or IL-8 (β=0.011; p=0.93) or IL-8 (0.01; 0.91). Country of origin (Iraq vs Syria) was not associated with mental health symptoms scores, IL-6, or Il-8 levels.

## Discussion

Our study of newly arrived refugees from Iraq and Syria in the United States found a positive association between war-related trauma exposure, measured using the well-established Harvard Trauma Questionnaire, and all three mental health outcomes, anxiety, depression, and PTSD symptoms. Importantly, supporting the hypothesis that pro-inflammatory interleukins might have differential effects on mental health, there was no significant association between IL-6 and IL-8. Interestingly, depression symptoms were inversely associated with IL-6, after controlling for age, gender, and war exposure. Regarding IL-8, adjusting for age in the linear regression model, war-related trauma exposure scores were not associated with IL-8, but anxiety scores were positively associated with IL-8.

There was no bivariate association between war-related trauma exposure and IL-6 or Il-8, even after adjusting for age, a risk factor for increased systemic inflammation [22,26,27]. However, when we also controlled for depression, pre-migration war-related trauma exposure was positively associated with IL-6, but not IL-8. This differs from the results of one study reporting no differences in IL-6 levels in war veterans (mean age=45.9 years) and healthy controls (mean age=47.2) [28]. However, when war veterans with PTSD were compared to a cohort of healthy elderly (aged 80 or older), IL-6 levels were significantly higher in the elderly, confirming the influence of age on neuroinflammation [28].

In a study of mental health disorders in children and adolescents, there was a positive association between depression and IL-6, unlike the negative association we found here. However, those findings were on younger participants where grouping was based on a clinical mental health diagnosis compared to healthy controls using the Diagnostic and Statistical Manual of Mental Disorders (DSM) IV criteria [29]. We used a validated self-reported scale to determine depression scores in our study. Moreover, the aforementioned study did not assess trauma exposure, another possible contributor to IL-6 levels based on our study's findings. It is well-recognized that a substantial proportion of children and adolescents have been exposed to adverse childhood experiences (ACEs) that might have similar effects on neuroinflammatory processes as war trauma [9,29]. This is in contrast to a study of PTSD severity in war veterans that found reduced IL-6 levels in those with higher PTSD severity [30].

The association we observed between IL-8 and anxiety is consistent with that reported in a study of non-war-exposed persons where IL-8 levels were higher in patients with various mental health disorders, including anxiety, depression, obsessive-compulsive disorder (OCD), and psychosis, compared to healthy controls [29]. However, our findings conflict with those from a study that reported that IL-8 was inversely associated with anxiety symptoms in suicidal patients [14]. Possible reasons for these study differences could be the age of study populations, where ours were older, prevalence of chronic diseases, and prior trauma exposures. The severity and duration of patients' conditions may also have differed between these studies. Our study did not specifically target suicidal patients [14]. The severity of mental health disorders in refugees versus non-war-exposed persons might also have contributed to observed differences in inflammatory biomarkers. Apart from being younger, another study assessed participants who had been hospitalized for various psychiatric conditions, whereas our study assessed participants who did not require hospitalization for mental illness at the time of the study [29]. Study participants originated from Iraq and Syria, respectively. However, there were no significant differences in terms of the associations between country of origin and mental health symptoms scores, neither in levels of IL-6 nor IL-8, respectively.

Strengths and limitations

Our study, like most prior studies on this topic, used a cross-sectional design so causal relationships cannot be determined. It would be ideal to relate changes in mental health symptoms to changes in inflammatory biomarkers over time. Some studies of non-refugee populations have reported an increase in inflammatory biomarkers after time has elapsed from the onset of their mental health symptoms, resulting in possible differences between our recently trauma-exposed population and other long-term studies [13]. Although the sample size was somewhat small, the power calculations revealed sufficient power to detect effect sizes for correlations around 0.4. Our participants also responded to a validated and comprehensive set of survey items that included validated scales for war-related trauma exposures and mental health symptoms.

One possible limitation is the lack of exclusion criteria in our study, especially regarding medical comorbidities. However, our inclusive approach adds to the generalizability of our study. Additionally, we controlled for other factors such as age, gender, and war exposure in our statistical analyses. However, it is possible that there was some sampling bias, in that refugees needed to seek information about the study voluntarily, although the recruitment process was free from any external pressure to participate.

We chose to focus on two common inflammatory biomarkers. However, other inflammatory biomarkers might have shown different results [28]. It is suggested that future studies should include a broader scope of inflammatory markers in refugees and their relationship to mental health and apply a prospective survey design to enhance our understanding of causal and temporal relationships.

Finally, another limitation is the inability to track changes in exposure, symptoms, and biomarkers over time. For example, displaced refugees have been potentially exposed to war trauma not only in their place of residence but also during their time in refugee camps prior to being cleared for refugee-immigration status to the United States. However, since systemic inflammatory processes due to trauma are likely to be rather slow processes, and we captured newly arrived refugees, we do not believe these factors markedly distorted findings.

## Conclusions

Study findings suggest that mental health symptoms secondary to war-related trauma exposure are associated with inflammatory biomarkers IL-6 and IL-8. However, based on current literature and the present study, the pathophysiological disease mechanisms involving IL-6 and IL-8 might differ in terms of trauma-associated mental health disorders. We demonstrated a differential association between mental health symptoms and these two inflammatory biomarkers. We found that depression was inversely associated with IL-6, whereas anxiety was positively associated with IL-8. Further investigation is needed in terms of the role of war-related trauma exposure and inflammatory processes as they relate to mental health disorders in refugees. Such knowledge is important to more effectively treat, prevent relapse, and eventually promote mental health in trauma-exposed and displaced refugees, sadly, a large and growing group of members of the international community.
